# Analysis of von Willebrand factor A domain-related protein (WARP) polymorphism in temperate and tropical *Plasmodium vivax *field isolates

**DOI:** 10.1186/1475-2875-8-137

**Published:** 2009-06-23

**Authors:** Saber Gholizadeh, Navid Dinparast Djadid, Hamid Reza Basseri, Sedigheh Zakeri, Hossein Ladoni

**Affiliations:** 1 Malaria and Vector Research Group (MVRG), Biotechnology Research Center (BRC), Pasteur Institute of Iran (PII), Tehran, Iran, Pasteur Avenue, PO BOX 1316943551, Tehran, Iran; 2 Department of Medical Entomology, School of Public Health, Tehran University of Medical Sciences, Tehran, Iran

## Abstract

**Background:**

The identification of key molecules is crucial for designing transmission-blocking vaccines (TBVs), among those ookinete micronemal proteins are candidate as a general class of malaria transmission-blocking targets. Here, the sequence analysis of an extra-cellular malaria protein expressed in ookinetes, named von Willebrand factor A domain-related protein (WARP), is reported in 91 *Plasmodium vivax *isolates circulating in different regions of Iran.

**Methods:**

Clinical isolates were collected from north temperate and southern tropical regions in Iran. Primers have been designed based on *P. vivax *sequence (ctg_6991) which amplified a fragment of about 1044 bp with no size variation. Direct sequencing of PCR products was used to determine polymorphism and further bioinformatics analysis in *P. vivax *sexual stage antigen, *pvwarp*.

**Results:**

Amplified *pvwarp *gene showed 886 bp in size, with no intron. BLAST analysis showed a similarity of 98–100% to *P. vivax *Sal-I strain; however, Iranian isolates had 2 bp mismatches in 247 and 531 positions that were non-synonymous substitution [T (ACT) to A (GCT) and R (AGA) to S (AGT)] in comparison with the Sal-I sequence.

**Conclusion:**

This study presents the first large-scale survey on *pvwarp *polymorphism in the world, which provides baseline data for developing WARP-based TBV against both temperate and tropical *P. vivax *isolates.

## Background

*Plasmodium vivax *is one of the two most prevalent species of human malaria parasites that occurs throughout the tropics, except in western and central sub-Saharan Africa, where the absence of Duffy factor on the surface of red blood cells largely protects the local populations [1]. In addition, recent studies have suggested that vivax malaria can become lethal in a similar way to severe falciparum malaria [2-5]. To date, the anti-malarial treatment and the vector control measures have not had a significant impact on the transmission of malaria from humans to mosquitoes. Therefore, vaccine-targeting antigens expressed on the surface of the sexual stages of malaria parasites, such as gametocytes, gametes, zygotes and ookinetes, are being considered for the development of a transmission-blocking vaccine (TBV) [6-8], a promising strategy for malaria control. The parasite has to undergo a complex development programme inside the mosquito from gametocyte to sporozoite [9]. So far, several studies have focused on the identification and characterization of TBV targets [10-14]. One of the TBV targets is a soluble protein that is called von Willebrand factor A domain-related protein (WARP), which is expressed in late ookinetes and early oocysts [14,15]. WARP could mediate ookinete attachment to the mosquito midgut, differentiation of ookinete to oocyst, and interactions with the mosquito basal lamina. Oocyst formation was reduced significantly when mosquitoes fed on an infected mouse passively immunized with the anti-WARP antibody. This indicates that the antibody interferes with WARP function by recognizing the protein on the surface of the parasite and makes it a candidate antigen for a TBV [14].

The malaria endemic area of Iran is located in the south-eastern part of the country, bordering Afghanistan, Pakistan, the Persian Gulf and the Oman Sea. This corner of Iran consists of Sistan and Baluchistan, Hormozgan and the tropical part of Kerman provinces, where malaria transmission has been found to be perennial, with *Anopheles stephensi*, *Anopheles culicifacies, Anopheles fluviatilis *and *Anopheles pulcherrimus *as the main vectors. More than 90% and 70% of the infections were due to *P. vivax *in the first and second peaks of transmission in 2007, respectively. In North, although malaria is under control since its re-emergence in 1994, *Anopheles maculipennis *and *Anopheles sacharovi *are the main vectors, while *Anopheles superpictus *and *Anopheles hyrcanus *are suspected as the secondary vectors.

Using asexual blood stages antigens, PvCSP and PvMSP-1, Zakeri *et al *[16,17] revealed the extent of genetic diversity in Iranian *P. vivax *populations. They also reported that both *csp *sequence types, VK210 and VK247, and the three allelic types of *msp-1 *(Belem, Sal-I and recombinant type) gene were identified among *P. vivax *populations [16,17]. Further, they reported limited sequence polymorphism in sexual stage antigens (*pvs25 *and *pvs28*) among field *P. vivax *populations in Iran [18]. Therefore, this investigation was designed to analyse the degree of polymorphism in the *warp *gene of *P. vivax *in low transmission areas in Iran by using sequence analysis. The rational explanation for high priority selection of this gene is: 1) the limited information on *P. vivax *sexual stage antigens in mosquito and their importance for TBV in the states under WHO Eastern Mediterranean Regional Office (EMRO) and 2) anti-WARP polyclonal antibody strongly inhibits (70–92%) *Plasmodium *development in the mosquito [14,19]. Moreover, this study will provide a baseline data for further applied studies including eventual field trials of experimental vaccines. On the other hand, despite the differences in vector composition and other epidemiological features in various endemic areas, this protein was reported to be highly conserved within and among different *Plasmodium *species [19]. Therefore, it is conceivable to use this TBV candidate of *P. vivax *for other *Plasmodium *species, such as *Plasmodium falciparum*. Furthermore, the present results would complement the available information regarding TBV candidate and would allow comparing and contrasting the Iranian *P. vivax *populations to those from different epidemiological settings. Therefore, to achieve this goal, first specific primers were designed to amplify the *pvwarp *gene and then characterize the gene structure among temperate and tropical Iranian *P. vivax *populations.

## Methods

### Study areas and sample collection

Samples were collected from symptomatic *P. vivax*-infected patients during 2000–2003 from the Ardebil province in the north of Iran (n = 31) and Sistan and Baluchistan province in the south (n = 60) during 2000–2006. In the North, malaria re-appeared after 20 years following a large displacement of people from the Republic of Azerbaijan and to some extent from Armenia in 1994; however, it came under control in northern Iran through a multi-disciplinary strategy in 2003. The transmission season is from June to October [17] and *P. vivax *is the only *Plasmodium *species detected microscopically. In addition, mixed *P. vivax *and *P. falciparum *infections were detected only by sensitive molecular methods in this region [20].

The second study areas are in the southern parts of Iran, including Sistan and Baluchistan bordering Afghanistan, Pakistan, the Persian Gulf and the Oman Sea. There are two peaks of malaria transmission in this area: the first, from May to August when *P. vivax *is the predominant species and the second, from October to November when both *P. vivax *and *P. falciparum *occur, sometimes in equal numbers.

All temperate and tropical *P. vivax *clinical isolates were diagnosed by light microscope examination of Giemsa-stained blood smear. The blood samples (1 ml) were collected on admission after informed consent was obtained from adults or from the parents or legal guardians of children. This study was approved by Ethical Review Committee of Pasteur Institute of Iran.

### The extraction of *P. vivax *DNA

*Plasmodium vivax *genomic DNA was extracted from the infected blood by standard phenol/phenol-chloroform extraction and ethanol precipitation as described by Snounou *et al *[21]. The DNA was dissolved in 30 μl of TE buffer (10 mM Tris-HCL, pH 8.0, 0.1 mM EDTA) and kept at -20°C until use.

### Primer designing

At the time of designing this study, the only available sequence for *pvwarp *gene in GeneBank was related to accession no. AB051630 (Tsuboi, direct submission). Therefore, the first set of primers was designed based on this sequence by using Gene Runner (version 3.05, 1994, Hastings Software Inc.) and BLAST [22] softwares. These newly desinged primers were used for amplification of *pvwarp *gene followed by cloning and sequencing of the amplified fragment. The sequencing results revealed that the first 50 amino acids of the outcoming sequence did not match the submitted sequence to GeneBank by Tsuboi. Thus, the second set of primers were designed from a 108 bp upsream based on sequence of *P. vivax *(ctg_6996) [23] as follow:

PvWF: 5'TAAGAGGGCAACACAAACG3'

PvWR: 5'ATCTTCACCTGCCCACTCC3'

Polymerase chain reaction (PCR) of *pvwarp *fragment was conducted by the above mentioned primers in all 91 Iranian *P. vivax *isolates. The reaction was carried out for 35 cycles at 95°C for 5 minutes, 95°C for 1 minute, 62°C for 1 minute and 72°C for 1 minute and a final primer extension at 72°C for 10 minutes.

### Molecular analysis of *pvwarp *gene

In order to define the extent of variability within *pvwarp*, the PCR products from 15 northern and 35 southern isolates were directly sequenced by using the designed primers. For this purpose, a ABI 3100 DNA sequencer (Kawsar, Biotech, Iran) was used. Nucleotide and amino acid sequences were aligned with the corresponding Sal-I sequence ([GenBank: XM-001608555]) by using MEGA4 [24] and CLUSTAL W [25]. Major alleles were classified based on protein sequence alignment and the tree was constructed with the neighbor-joining method, Kimura two-parameter and pairwise deletion, based on amino acid sequences of PvWARP from Iranian isolates and other *Plasmodiun *species in GenBank.

To identify B-cell epitope binding sites and secondary structure in two groups, further bioinformatic analysis was done on amino acid sequences of PvWARP protein by using B-cell epitope prediction [26] and Jemboss [27] softwares. Nucleotide sequences are available in the GenBank, European Molecular Biology Laboratory (EMBL) and DNA Data Bank of Japan (DDBJ) databases under [GenBank: FJ170289 to FJ170338].

## Results

**Figure 1:**
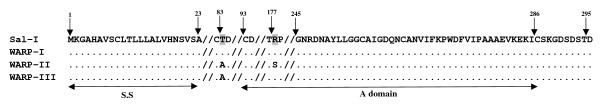
**Amino acid sequence alignment of the three *pvwarp *variants in 50 Iranian *P. vivax *isolates**. The sequences were compared with Sal-I ([GenBank: XM-001608555] and represent the secretary signal sequence (SS), the A domain. The representative PvWARP haplotypes are: PvWARP-I (T/R, 8%), PvWARP-II (A/S, 88%) and PvWARP-III (A/R, 4%). //, indicates conserved sequences and dots represent identical residues.

In the primary phase of this study, *pvwarp *was sequenced by using designed primers based on the only available reference sequence (AB051630). The obtained 886 bp sequences from Iranian *P. vivax *isolates were aligned with that reference ([GenBank: AB051630]) reported by Tsuboi, and also with Salvador-I sequence ([GenBank: XM-001608555]), which showed 98% and 99% similarity, respectively. In the second phase, because the sequencing results revealed that the first 50 bp of the obtained sequences did not match the submitted sequence to GeneBank, a new pair of primers (PvWF and PvWR) were designed from a 108 bp upsream based on sequences of *P. vivax *(ctg_6991) [23]. These primers amplified a fragment of about 1044 bp in 31 temperate northern and 60 tropical southern isolates from Iran, with no size polymorphism. Sequencing the target amplified fragment showed that this gene contains a 886 bp open reading frame encoding a putative 295 amino acid protein with a calculated molecular mass of ~32.2 kDa. The analysis of the primary structure by SignalP [28] indicates that the first 69 bp of nucleotides (23 amino acids) are signal sequences, and the remaining sequences from amino acids 93–286 contain a von Willebrand factor type A module like domain (domain A) (Figure 1).

Finally, based on the sequencing result, 15 northern and 35 southern *P. vivax *isolates were selected randomly for sequencing analysis and the results revealed three distinct variants among the 50 sequenced samples (Figure 1). Two isolates from each study area showed 100% similarity to Sal-I sequence ([GenBank: XM-001608555]), while the majority (13 isolates from north and 23 isolates from south) had 99% homology with Sal-I isolate (Table 1). Based on nucleotide analysis, in *pvwarp *gene, four substitutions at positions 102, 222, 247 and 531 were detected.

**Table 1:** Comparison of *csp*, *msp-1 *and *warp *sequence types in Iranian *P. vivax *parasite populations

	**Temperate region (North)**	**Tropical region (South)**
*csp *[16]	VK210 (99.5%)	VK210 (70.5%)
	VK247 (0.5%)	VK247 (17.5%)
		Mix (12%)

*msp 1 *[17]	Type 1 (Belem) (75%)	Type 1 (Belem) (17.2%)
	Type 2 (Sal I) (21%)	Type 2 (Sal I) (56.3%)
	Type 3 (Recombinant) (4%)	Type 3 (Recombinant) (26.5%)

*warp *(present study)	Type 1 (Sal I) (11.8%)	Type 1 (Sal I) (6%)
	Type 2 (88.2)	Type 2 (88%)
	Type 3 (-)	Type 3 (6%)

*Anopheles *vectors [16,17]		*An. stephensi*
	*An. maculipennis*	*An. fluviatilis*
	*An. sacharovi*	*An. culicifacies*
		*An. pulcherrimus*

**Figure 2:**
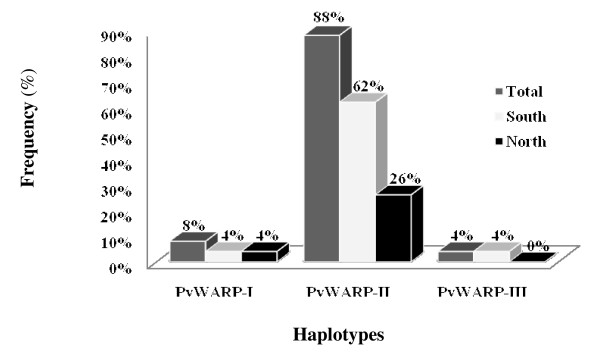
**Frequency distribution of different PvWARP haplotypes obtained from 50 *P. vivax *samples collected from north and south of Iran**. PvWARP haplotypes are: PvWARP-I (T/R, 8%), PvWARP-II (A/S, 88%) and PvWARP-III (A/R, 4%).

In Sal-I sequence ([GenBank: XM-001608555]), amino acids at residues of 83 and 177 are T (ACT), and R (AGA). However, in 13 isolates from north and 21 isolates from south, those positions substituted with A (GCT) and S (AGT), respectively, and in two remaining isolates from south, only one substitution at position 83 was observed in comparison with Sal-I (substitution of T to A) (Figure 1). In addition, PvWARP has two synonymous and two non-synonymous substitutions in amino acid sequence in northern and southern isolates from Iran. Further analysis of amino acid sequences with B-cell epitope prediction software showed that non-synonymous substitutions are not in epitope sites, and the analysis of protein secondary structure in two groups showed that non-synonymous substitutions have not changed the structure of PvWARP. Total frequency of the three PvWARP haplotypes (I, II and III) in examined samples are (T/R, 8%), (A/S, 88%) and (A/R, 4%) respectively (Figure 2).

**Figure 3:**
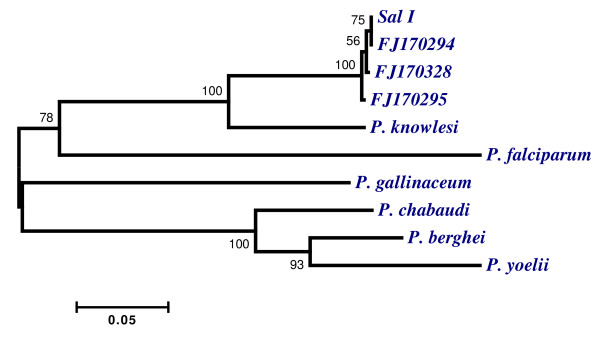
**Phylogenetic tree constructed with MEGA4 program based on the amino acid sequence of WARP gene in *Plasmidum *species**. [GenBank: FJ170294] is the represantative isolate from PvWARP-I haplotype, that is 100% similar to Sal-I ([GenBank: FJ170314 and FJ170318 from south and FJ170297 from north]). [GenBank: FJ170295] is the representative isolate from PvWARP-II haplotype that have two non-synonymouse amino acids ([GenBank: FJ170289–FJ170303 from north and FJ170304-FJ170338 from south]). [GenBank: FJ170328] is the representative isolate from PvWARP-III haplotype ([GenBank: AJ170330 from south]) that has one non-synonymous amino acid sequence. The previously published sequences are Sal-I ([GenBank: XM-001608555]), *P. knowlesi *([GenBank: AM910983]), *P. falciparum *([GenBank: XM 001349468]), *P. gallinaceum *[19], *P. chabaudi *([GenBank: XM 739938]), *P. berghei *([GenBank: AY247951]) and *P. yoelii *([GenBank: XM 723529]).

Phylogenetic tree constructed based on the PvWARP sequences originated from this study revealed that 50 sequences were divided into three distinct haplotypes. The first haplotype includes Sal- I and sequences derived from the present study, the second one includes sequences that contain two non-synonymous substitutions, and the last one includes sequences that contain one non-synonymous substitution in comparison with Sal-I strain. *Plasmodium knowlesi *is the nearest taxa to *P. vivax*, while *P. falciparum*, *Plasmodium chabaudi*, *Plasmodium berghei*, *Plasmodium yoelii *and *Plasmodium gallinaceum *stand at farther distance from *P. vivax *(Figure 3).

## Discussion

*Plasmodium vivax *remains a significant public health problem in parts of Latin America and Asia, where it can account for 40–90% of malaria cases. In addressing the developing a vaccine for *P. vivax *malaria, understanding the epidemiology of *P. vivax *and the polymorphism of different vaccine candidate antigens at sexual and asexual stages is highly needed. Recent advances increase confidence that a mosquito stage transmission-blocking malaria vaccine will be feasible [29]. For identifying malaria TBV targets, most strategies have been focused on gametocytes, gametes or zygotes [7,30].

Little is known about the mechanisms that direct parasite development to its mosquito host. More recently, some plasmodial proteins have been identified as potential antigens for a mosquito-stage transmission-blocking vaccine, including chitinase [19,31,32], CTRP [19,33-35], secreted ookinete adhesive protein (SOAP) [36], membrane-attack ookinete protein (MOAP) [37], WARP [19] and lectin adhesive-like protein (LAP)[38,39]. In mosquito infectivity, an important role has been shown for each of these proteins through knockout experiments, but their utility for mosquito stage vaccine is still unclear [29]. WARP, a gene encoding a *Plasmodium *surface protein with a von Willebrand factor A like adhesive domain, is expressed only in late ookinetes and early oocysts [15]. The study conducted by Li *et al *[19] showed that anti-*P. falciparum *WARP significantly reduced the infectivity of *P. gallinaceum *to *Aedes aegypti *and *P. falciparum to Anopheles *mosquitoes. On the other hand, Richards *et al *[40] reported a limited polymorphism in PfWARP within clinical isolates collected from a wide variety of geographical regions. They showed three non-synonymous substitutions in positions 140 (F to L), 148 (V to F/L) and 228 (T to A) in comparison to *P. falciparum *3D7 strain sequence [40]. However, so far, there was not any published data on polymorphism of WARP of *P. vivax *field isolates. Therefore, this investigation was designed to analyse the sequence of WARP in 50 field isolates from two different malaria settings in north (re-emerged) and south (endemic) of Iran.

The result revealed a limited polymorphism in *pvwarp *gene of *P. vivax *field isolates in low transmission malaria settings with different vector composition. Nucleotide sequence analysis showed that these isolates have polymorphisms in four positions 102, 222, 247 and 531 with only two non-synonymous substitutions at residues 83 (T to A) and 177 (R to S). However, these polymorphic positions in *P. vivax *are not homologous to those reported for the *P. falciparum *gene (positions 140, 148 and 228). Furthermore, the comparison of Iranian isolates with AB051630 by BLAST (direct submition by Tsuboi in 2001) showed a similarity of 98%. However, the [GenBank: AB051630] in the first 50 amino acid residues dose not match the recently published Sal-I gene sequence ([GenBank: XM-001608555]). This may be due to the misread or sequencing error in the original study. Having all reading frames, the correct amino acids could be detected by looking at frameshift against Sal-I sequence. In this regard, it might be required to revise the AB051630 sequence in the GenBank.

The secondary structure of deduced amino acid sequence of PvWARP was analysed by using Jemboss software [27]. In comparison with Sal-I strain, the two non-synonymous positions (aa. 83 and 177), that are located in β-sheet and coil region, did not change the protein configuration in three detected haplotypes within 50 sequenced samples, indicating the conserved nature of this gene in Iranian isolates.

The outcoming results from 50 sequences addressed for the first time the sequence diversity in PvWARP from vivax endemic region in the Middle East. In spite of detecting two and three haplotypes in temperate northern and tropical southern isolates of Iran based on their frequency distribution (Figure 2), the majority of the isolates were categorized in two types: type 1 was 100% similar to Sal-I strain and type 2 had two non-synonymous substitutions at amino acid residues 83 and 177. Southern isolates had one more type (type 3) that contains a non-synonymous substitution in comparison with Sal-I strain (Figure 1). The present results were in parallel to the findings reported by Richards *et al *[40], in which limited polymorphism (three positions) was detected in PfWARP within 19 different geographical fields and three laboratory strains.

In addition, phylogenetic tree were constructed based on PvWARP amino acid sequences from the present study and from different *Plasmodium *species available in GenBank. The high similarity (61%) among PvWARP and PfWARP sequences at amino acid level suggests significant conservation of WARP primary structure among these two distinct *Plasmodium *species. As mentioned by Yuda *et al *[15], it is assumed that the common invasion mechanism may widely exist throughout the *Plasmodium *parasites. This finding was also supported by the work carried out by Li *et al *[19], in which the sera produced against PfWARP significantly reduced the infectivity of PgWARP to *Aedes aegypti*. Based on these findings, it is postulated that theoretically, WARP can be used as an universal TBV against mixed *P. vivax *and *P. falciparum*. However, it should be noted that it is not clear whether the antibody-binding sites for WARP, which play a role in differentiation of ookinete to oocyst in mosquito midgut, are located within the identical amino acid regions of both species.

On the other hand, *pvmsp-1 *and *pvcsp *genes are two vaccine candidates for blood stage of malaria infection. Zakeri *et al *[16,17] reported a *csp *genetic diversity among temperate and tropical *P. vivax *isolates from Iran. Parasites collected in the northern area were almost exclusively of the VK210 (99.5%) type, while both VK210 (70.5%) and VK247 (17.5%) types were present in the southeastern areas. Among sequenced isolates in the present study, we did not detect any correlation between the *pvwarp *haplotypes and the type of either *pvcsp *or *pvmsp-1 *because these three haplotypes were distributed among both three haplotypes of *pvwarp *(Table 1). However, this is not consistent with the findings of this study showing the presence of different *Anopheles* vector species, zoogeography, ecology and vectorial capacity in the study areas. This may point the fact that the polymorphisms are not selected/correlated with transmission by different vector species in two different malaria settings of Iran. This provides an advantage for the wider use of proposed WARP-based transmission-blocking vaccine. Furthurmore, although the endemicity is low in both areas in the south, malaria has never been interrupted, while the northern areas were malaria-free for a period of more than 30 years till 1994.

## Conclusion

In conclusion, this study presents the first large-scale survey on PvWARP polymorphism in the world, that provides a baseline data for developing WARP-based TBV against both temparate and tropical *P. vivax *isolates. So far, the polymorphisms in the few proteins of sporogony cycle of malaria parasites studied. Low degree of the polymorphism in Iranian PvWARP state that the proteins expressed in the mosquito stages appear to be less polymorphic than those expressed in the blood stages, which might indicate that the selective pressure in the mosquito is less strong than that in the mammalian host. Accordingly, limitted polymorphism in PfWARP and PvWARP sequences seems to be useful for TBV studies in the oriental corner of EMRO, including Iran, Pakistan and Afghanistan. Further experimental work is under progress in to define the transmission blocking activity of anti-WARP antibodies to disrupting the development of ookinete to oocyst within the mosquito vectors.

## Competing interests

The authors declare that they have no competing interests.

## Authors' contributions

SZ designed the study, supervised the sequence analysis and jointly finalized the manuscript. NDD coordinated the project, participated in data analysis and jointly finalized the manuscript. HRB and HL contributed in data collection and administrative coordination, SG carried out the molecular genetic studies, sequence analysis and drafted the manuscript. All authors read and approved the the final manuscript.
